# The Reformed Public House

**Published:** 1924-03

**Authors:** J. Douglas Edwards


### The Reformed Public House.

SIR,—I have read the correspondence on this question with interest, but, while agreeing with much that you say in your editorial remarks, I should like to point out that so long as the present licensing system continues, we are not likely to secure the reformed public house which you and many of your readers desire. It is not to be expected that the large brewery companies, whose main objective is the maintenance of large dividends, will encourage the universal adoption of reforms which tend to reduce consumption. I know that they ought to provide decent houses, but they don't. Something may be done here and there, but in the main it will be where the facilities for the sale of alcoholic liquors are likely to develop.

The State monopoly, as adopted in the Carlisle system, on the other hand removes the incentive of private profit, provides the reformed public house and, at the same time, secures substantial reforms which are impossible by any other means. It allows of the closing of redundant licences, and of the grocers' licence. It ensures substantial administrative economies. The Public House Trust Companies and the People's Refreshment House Association were the pioneers; they worked, necessarily, on restricted lines. But the liquor problem is really one of the towns, for 80 per cent. of the people of this land are town dwellers and so the treatment of the question on Carlisle lines, with all respect, seems to me to be the real method of advance.

J. Douglas Edwards.

